# Sex Differences in the Prevalence and Modulators of Sleep-Disordered Breathing in Outpatients with Type 2 Diabetes

**DOI:** 10.1155/2018/7617524

**Published:** 2018-04-01

**Authors:** T. Kroner, M. Arzt, M. Rheinberger, M. Gorski, I. M. Heid, C. A. Böger, S. Stadler

**Affiliations:** ^1^Department of Internal Medicine II, University Hospital Regensburg, Franz-Josef-Strauss-Allee 11, 93053 Regensburg, Germany; ^2^Department of Nephrology, University Hospital Regensburg, Franz-Josef-Strauss-Allee 11, 93053 Regensburg, Germany; ^3^Department of Genetic Epidemiology, University of Regensburg, Franz-Josef-Strauss-Allee 11, 93053 Regensburg, Germany

## Abstract

In patients with type 2 diabetes, sleep-disordered breathing is a widespread cause of deteriorated quality of life. However, robust prevalence estimates for sleep-disordered breathing in patients with type 2 diabetes are limited due to scarce data. We investigated sex differences in sleep-disordered breathing prevalence and its modulators in the DIACORE SDB substudy, a sample of outpatient type 2 diabetes. 721 participants were tested for sleep-disordered breathing using a two-channel sleep apnoea monitoring device. Patients were stratified according to the severity of sleep-disordered breathing, defined as an apnoea-hypopnoea index < 15, ≥15 to 29, and ≥30 events per hour as no/mild, moderate, and severe sleep-disordered breathing, respectively. In the 679 analysed patients (39% women, age 66 ± 9 years, body mass index 31.0 ± 5.4 kg/m^2^), the prevalence of sleep-disordered breathing was 34%. The prevalence of sleep-disordered breathing was higher in men than in women (41% versus 22%, *p* < 0.001) and increased with age (15%, 21%, and 30% in women and 35%, 40%, and 47% in men in those aged 18–59, 60–69, or ≥70, respectively; age trend *p* = 0.064 in women and *p* = 0.15 in men). In linear regression analysis, age, BMI, and waist-hip ratio were associated with apnoea-hypopnoea index. Modulators for higher apnoea-hypopnoea index seem to be similar in men and women.

## 1. Introduction

The International Diabetes Federation reports a worldwide prevalence of diabetes mellitus of 8.8%, where type 2 diabetes (T2D) makes up 87–91% in high-income countries and is more common in men than in women [1]. Because of the aging population and the increasing number of obese people, the prevalence of T2D has become more than doubled during the last three decades [2]. T2D may lead to several late diabetic comorbidities like neuropathy, nephropathy, and arteriosclerosis.

Sleep-disordered breathing (SDB), which is classified into obstructive and central sleep apnoea, is characterised by repetitive apnoeas and hypopnoeas, arousals from sleep as well as alterations of heart rate, and elevated blood pressure [3, 4]. Using an apnoea-hypopnoea index (AHI) cut-off of ≥15/h, the prevalence of SDB ranges from 7 to 14% in men and 2 to 7% in women, with increases over the last two decades [5, 6].

It is well documented that treatment with positive airway pressure effectively suppresses obstructive sleep apnoea, reduces sleep fragmentation, and restores normal sleep structure in patients with cardiac disease [7, 8]. It leads to significant improvement of daytime sleepiness and quality of life, even in patients reporting only mild SDB-related symptoms [9]. Previous studies investigated the association between SDB and T2D. When focusing on moderate to severe forms of SDB (AHI ≥ 15/h) with an indication for treatment [4], 24 to 53% of patients with T2D suffer from SDB [10–12], compared to 16% without T2D [10].

However, previous studies provide only limited information about the prevalence and its modulators for SDB in women with diabetes. Even if there was a nearly balanced ratio in the number of men and women in most studies, they used smaller sample sizes [10–12] or included only selected patients such as obese [12] or hospitalized patients [13]. Both T2D and SDB are common in men and women, but often underdiagnosed in women [14, 15]. We analysed a sample of patients with T2D with an equal number of men and women (61%/*n* = 412 men and 39%/*n* = 267 women) and investigated sex differences in the prevalence of SDB as well as modulators that were associated with SDB.

## 2. Material and Methods

### 2.1. Study Design

The investigated patients were participants of the DIACORE (DIAbetes COhoRtE) SDB substudy [16]. DIACORE is designed as a two-center, prospectively planned study of T2D patients of European descent, with a baseline survey conducted in 2010–2014 and recruitment and ascertainment described previously [16, 17]. Briefly, written invitations were mailed to all T2D patients registered with five medical insurance companies in the respective year of the mailing and to all T2D outpatients of two diabetologists in Regensburg that had visited the offices within the last 6 months of the mailing. Further, invitations were sent to T2D patients who had received inpatient treatment at the University Hospital Regensburg's Internal Medicine Departments within two years prior to the mailing. Overall, 4226 patients contacted DIACORE, of which 1226 did not fulfil the inclusion and exclusion criteria. Diabetes status was ascertained by assessing diabetes medication or by validating self-report. Patients were subjected to a standardized online questionnaire, blood sampling, and physical examination at the two study centers.

Of 1036 individuals invited to participate in the DIACORE SDB substudy, 721 individuals with T2D from the region around Regensburg, Germany, agreed to participate and were tested with a two-channel ambulatory SDB monitoring device (ApneaLink®, ResMed). In 679 patients (94% of the 721 tested), complete SDB parameters were recorded. Apparative monitoring for SDB was not performed in the Mannheim study center. A two-year follow-up is currently ongoing [17]; for this investigation, the cross-sectional baseline dataset was used. The protocol, data protection strategy, and study procedures were approved by the ethics committees of both participating institutions and are in accordance with the Declaration of Helsinki. Patients participated in DIACORE only after providing informed written consent.

### 2.2. Study Population

All T2D outpatients inhabiting the city and county of Regensburg or Speyer were eligible for DIACORE. Further inclusion criteria were the ability to fully understand the study information and to provide written informed consent, age ≥ 18 years, and self-reported Caucasian ethnicity. Exclusion criteria were chronic renal replacement therapy (haemodialysis, peritoneal dialysis, or transplantation), history of active malignancy within the last five years, autoimmune-disease potentially affecting kidney function, hemochromatosis, known pancreoprivic or self-reported type 1 diabetes, acute infection, fever, pregnancy, and chronic viral hepatitis or HIV-infection. For the DIACORE SDB substudy, patients were included if they consented to perform SDB monitoring and excluded if they currently use positive airway pressure therapy.

### 2.3. Assessment of SDB

Nasal flow and pulse oximetry were measured using the ApneaLink device (ResMed, Sydney, Australia), which has been validated in several studies for testing of SDB as described previously [18, 19] and is easy to mount at home by the patient himself. Trained study personnel instructed participants in the use of the device in a standardized fashion. Comparing ApneaLink to the gold standard polysomnography, studies have reported a sensitivity of 73–94% and a specificity of 85–95% using an AHI cut-off value of 15/h [19, 20]. AHI, oxygen desaturation index (ODI), mean oxygen saturation (SpO_2_), and minimum SpO_2_ were documented. The default settings of the monitoring device were used for the definitions of apnoea, hypopnoea, and desaturation: apnoea was defined as a ≥ 80% decrease in airflow for ≥10 seconds; hypopnoea was defined as a decrease in airflow by 50–80% versus baseline for ≥10 seconds followed by a ≥ 4% decrease in oxygen saturation.

No or mild SDB was defined as AHI < 15/h, moderate SDB as AHI ≥ 15/h to 29/h, and severe SDB was defined as AHI ≥ 30/h. Periodic breathing (Cheyne-Stokes respiration) was detected by automatic pattern recognition [21]. Due to the lack of a chest band, a clear differentiation between types of SDB, such as obstructive and central apnoea, was not possible.

Additionally, subjective daytime sleepiness was assessed using the self-administered validated Epworth Sleepiness Scale (ESS). Individuals were asked to rate their likelihood of falling asleep in several common situations. Scores range from 0 (least sleepy) to 24 (sleepiest). Excessive daytime sleepiness is defined as a score of 11 or higher [22].

### 2.4. Statistical Analysis

Descriptive data are presented as absolute and relative numbers or as mean ± standard deviation (SD). Normally distributed values of baseline characteristics were evaluated with analysis of variance (ANOVA). Differences in AHI between age groups were tested with ANOVA and followed by pair-wise *t*-tests (Bonferroni corrected level of significance); sex differences in AHI were tested with *t*-tests.

Linear regression models were used for continuous variables and logistic regression for categorical variables, in order to investigate the association between different clinical variables. Sex, age, body mass index (BMI), waist-hip ratio, systolic blood pressure, and duration of T2D were included as covariates in these models. Variables included in the linear regression models were either normally distributed or transformed prior to incorporation in the models. Results are given as regression coefficient *B* in linear models and as odds ratio OR in logistic regression models with 95% confidence interval (CI); *p* values less than 0.05 were considered statistically significant. Data were analysed using the SPSS statistical software package (SPSS 20.0, IBM SPSS Statistics, Armonk, New York, USA).

## 3. Results

### 3.1. Patient Characteristics

Of the 721 patients with T2D that were tested for SDB, 42 patients had incomplete data recordings and could not be analysed (online Supplement Figure S1). Of these, 679 participants were entered into the analysis, of which 39% (*n* = 267) were women. Patients were 66 ± 9 years old, and BMI was 31.0 ± 5.4 kg/m^2^. Their glycated haemoglobin (HbA_1c_) was 6.8 ± 1.1%, and the average duration of T2D was 10.1 ± 8.0 years (Table 1). Women were of about the same age compared to men, but had a higher BMI (*p* < 0.001) and lower diabetes duration (*p* = 0.057) than men.

Patients with severe SDB were older, predominantly male, and had a significant higher BMI as well as waist-hip ratio compared to patients with no/mild SDB (*p* = 0.088, *p* = 0.002, *p* < 0.001, and *p* < 0.001, resp.) (Table 2). Patients with severe SDB had also a significant larger waist circumference (*p* < 0.001). They showed lower high-density lipoprotein cholesterol (HDL-C) as well as higher systolic blood pressure, higher triglyceride levels, and more severe insulin resistance quantified by homeostasis model assessment (HOMA-IR [23]), but these values were not statistically significant. HbA_1c_ levels and T2D duration were comparable. Administration of incretin may have implications on prognosis for myocardial infarction in patients with T2D [24, 25]. In our study, there was no difference in incretin administration between women and men (Table 1) as well as between different severities of SDB (Table 2).

### 3.2. SDB Parameters

**Figure 1:**
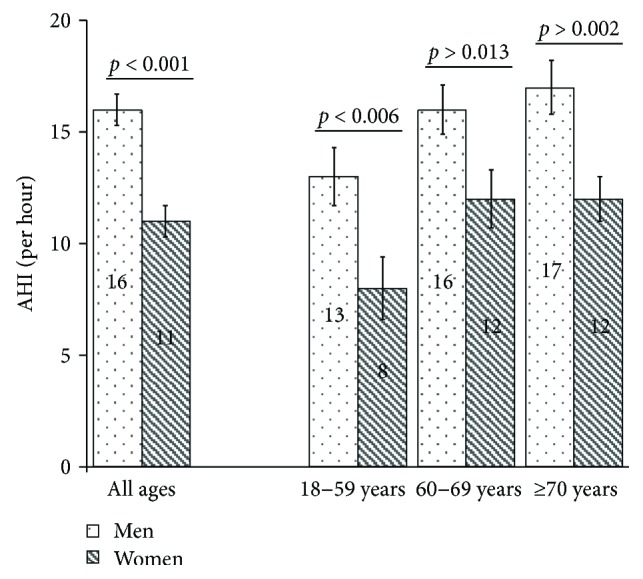
Apnoea-hypopnoea index (AHI) in men and women of the total sample (*n* = 679) and of different age groups (18–59, 60–69, and ≥70 years) (*n* = 164, *n* = 286, and *n* = 229), stating mean values in the bar and including standard errors and *p* values from *t*-tests. AHI was significantly higher in men than in women in the whole sample as well as in different age groups.

Table 2 presents the different SDB parameters and symptoms. ODI increased with higher severity of SDB. AHI was significantly higher in men than in women (mean 16 versus 11 per hour, resp., *p* < 0.001), also when patients were divided into different age groups (Figure 1). Additionally, AHI increased between the different age groups (*p* = 0.102 in men, *p* = 0.045 in women, and *p* = 0.006 overall, tested by ANOVA). Furthermore, patients with moderate or severe SDB spent more than 2 hours with SpO_2_ < 90% (Table 2).

### 3.3. The Prevalence of SDB in Patients with T2D

**Figure 2:**
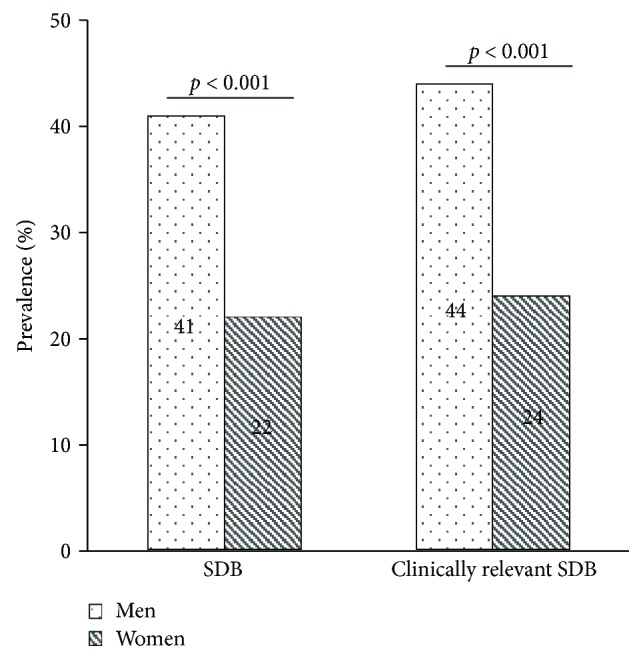
Bar charts showing the prevalence of sleep-disordered breathing (SDB) and clinically relevant SDB (AHI ≥5/h + ESS ≥ 11 or AHI ≥ 15/h) in 412 men and 267 women (%); *p* values assessed by chi-square tests. *n* = 679. SDB as well as clinically relevant SDB was significantly more present in men than in women.

**Figure 3:**
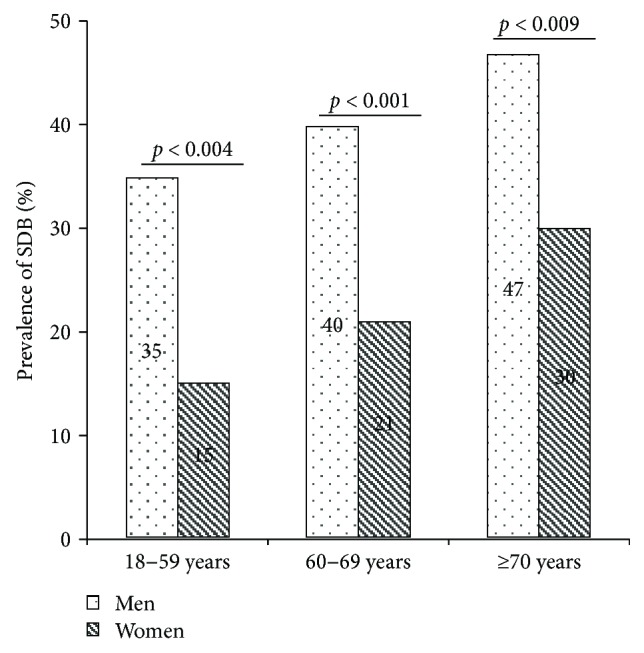
Bar charts with the prevalence of sleep-disordered breathing (SDB) in men and women in different age groups (%); *p* values were assessed by chi-square tests. *n* = 679. SDB was significantly more present in men than in women in each age group.

The prevalence of SDB in our study sample was 22% in women, 41% in men, and 34% overall. The prevalence of SDB was significantly higher in men compared to women overall (*p* < 0.001) (Figure 2) and in each age group (*p* = 0.004, *p* = 0.001, and *p* = 0.009) (Figure 3). Using an alternative definition of clinically relevant SDB (AHI ≥ 5/h + ESS ≥ 11 or AHI ≥ 15/h) led to similar results (44% in men, 24% in women, and 36% overall; *p* < 0.001) (Figure 2). The prevalence of SDB in both men and women increased with age, but this was statistically only significant in the whole sample (*p* = 0.151 in men; *p* = 0.069 in women; and *p* = 0.014 in either sex, tested by logistic regression analysis) (Figure 3).

### 3.4. Modulators Associated with SDB in Patients with Type 2 Diabetes

Using logistic regression by sex, age ≥ 70 years, BMI ≥ 30 kg/m^2^, waist-hip ratio ≥ 0.85 in women and ≥0.9 in men [27] as well as systolic blood pressure ≥ 130 mmHg, and T2D duration were analysed as covariates all in one model to investigate an association with SDB (AHI ≥ 15/h). In women, age ≥ 70 years (OR 1.95, 95% CI [1.04, 3.66], *p* = 0.039) and systolic blood pressure ≥ 130 mmHg (OR 2.09, 95% CI [1.09, 4.02], *p* = 0.026) were significantly and independently associated with SDB. BMI ≥ 30 kg/m^2^_,_ waist-hip ratio ≥ 0.85, and T2D duration were not significantly associated with SDB (OR 1.56 [0.82, 2.97], *p* = 0.175; OR 2.64 [0.97, 7.14], *p* = 0.057; and OR 0.99 [0.95, 1.04], *p* = 0.733, resp.).

In men, BMI ≥ 30 kg/m^2^ (OR 2.27, 95% CI [1.49, 3.47], *p* < 0.001) and T2D duration (per year; OR 1.03, 95% CI [1.00, 1.05], *p* = 0.033) were significant and independent modulators were associated with SDB. Age ≥ 70 years, waist-hip ratio ≥ 0.9, and systolic blood pressure ≥ 130 mmHg were not significantly associated with SDB in men (OR 1.52 [0.97, 2.37], *p* = 0.056; OR 1.05 [0.37, 2.96], *p* = 0.929; and OR 1.10 [0.70, 1.73], *p* = 0.676, resp.).

Using a general regression model including both sexes with sex, age ≥ 70 years, BMI ≥ 30 kg/m^2^, waist-hip ratio ≥ 0.85, systolic blood pressure ≥ 130 mmHg, and T2D duration as covariates, besides age ≥ 70 years (OR 1.65, 95% CI 1.2, 2.4, *p* = 0.007) and BMI ≥ 30 kg/m^2^ (OR 2.08, 95% CI 1.5, 3.0, *p* < 0.001), only male sex was independently associated with SDB (OR 2.57, 95% CI 1.7, 3.8, *p* < 0.001). WHR ≥ 0.85, systolic blood pressure ≥ 130 mmHg, and T2D duration were not significantly associated with SDB in either sex (OR 1.87, 95% CI [0.8, 4.4], *p* = 0.156; OR 1.37, 95% CI [1.0, 2.0], *p* = 0.091; and OR 1.02, 95% CI [0.99, 1.04], *p* = 0.106, resp.).

In regression models with all covariates listed above plus including interaction terms of each of the other covariates with sex, no interaction with sex was detected (interaction terms for age ≥ 70 years, BMI ≥ 30 kg/m^2^, WHR ≥ 0.85, systolic blood pressure ≥ 130 mmHg, and T2D duration: *p* = 0.527, *p* = 0.333, *p* = 0.999, *p* = 0.128, and *p* = 0.280, resp.). Thus, modulators for SDB seem to be similar in men and women.

### 3.5. Modulators Associated with AHI in Patients with Type 2 Diabetes

Linear regression analysis (Table 3) showed that a higher AHI was independently associated with higher age, higher BMI, and higher waist-hip ratio in women. In men, only age and BMI, but not waist-hip ratio, were significantly associated with AHI.

In the entire sample, male sex, age, BMI, and waist-hip ratio were associated with a higher AHI independent of the other covariates. Systolic blood pressure and T2D duration showed no significant association with AHI in either sex.

Adding an interaction term of waist-hip ratio with sex to the regression model stated above, no interaction with sex was detected (interaction *p* = 0.503). Thus, modulators for a higher AHI seem to be similar in men and women.

Both, metabolic syndrome and SDB are known to promote cardiovascular disease and arrhythmia [28–30]. Due to this, we added metabolic syndrome to the multivariable analysis and it appeared as a significant modulator of AHI in the whole sample (*B* = 5.139; 95% CI 2.913, 7.364; *p* < 0.001) as well as in women (*B* = 5.099; 95% CI 1.422, 8.776; *p* = 0.007) and in men (*B* = 5.186; 95% CI 2.350, 8.022; *p* < 0.001).

## 4. Discussion

The present study investigating SDB in 679 patients with T2D obtained several key findings. First, moderate to severe SDB, defined as AHI ≥ 15/h, is present in more than a third of our patients with T2D. Second, the prevalence of moderate to severe SDB increases with age and is more common in men than in women yielding a prevalence of 40% and 21% in our sample of 60 to 69-year-old men or women, respectively. Third, age, BMI, and waist-hip ratio are significant and independent modulators were associated with higher AHI. There were no significant differences of these effects between men and women.

The prevalence of SDB in community samples was modestly lower than in samples of patients with T2D. In obese participants of the same age group with a BMI (30.0 to 39.9 kg/m^2^) as seen in our sample, 29% of men and 14% of women have SDB [5].

Our results of 34% of patients with T2D with SDB are consistent with the seven prior studies including more than 150 patients with T2D and using polysomnography or SDB monitoring devices for diagnosis of SDB (Table 4). Differences in prevalence could be explained by differences in participant recruiting, patient characteristics, definitions of sleep apnoea, or methods of monitoring for SDB (Table 4 and online Supplement Table S2).

Some studies showed higher prevalence estimates than our study [12, 31] (Table 4). The reason for these higher prevalence estimates might be explained by a higher BMI of participants (Foster et al. BMI = 36.5 ± 5.8 kg/m^2^, Schober et al. BMI = 32.6 ± 6.7 kg/m^2^, and BMI = 31.0 ± 5.4 kg/m^2^ in the DIACORE SDB substudy) [12, 31] since obesity is a known risk factor for SDB [1, 32, 33].

Other studies showed lower prevalence estimates. The patient populations of those studies had significantly lower BMI (25.1 ± 3.6 kg/m^2^) [34] or were significantly younger [10, 13].

Similar to previous studies [31, 35], we found a higher prevalence of SDB in men than in women.

In previous studies, varied information about the modulators of SDB in patients with diabetes has been presented. Lam et al. found that male sex, higher age as well as BMI, and diastolic blood pressure were independently associated with AHI [35]. In the present study, age, BMI, and waist-hip ratio were significantly associated with AHI.

Even if there were no significant differences in modulators for a higher AHI between the sexes, waist-hip ratio showed an association with AHI in women in the DIACORE substudy. This finding might be explained by the differences in the distribution of bodily fat between the sexes. In women, fat accumulation in the hip region is more common compared to men [36]. Therefore, it is possible that the effect of a pathologically increased waist-hip ratio on AHI could be higher in women than in men.

There is evidence that diabetes and its level of severity could contribute to the development of SDB and its worsening [38, 39]. Although this was only a cross-sectional analysis, our data do not support this hypothesis, since HbA_1c_, HOMA-IR, and the duration of diabetes were not associated with the presence or severity of SDB.

On the other hand, there is evidence that SDB may contribute to impaired glucose metabolism [40]. Studies investigating the effects of treatment of obstructive sleep apnoea with continuous positive airway pressure show different results. In a randomized controlled trial, this therapy significantly improved glucose tolerance [41]. However, other studies did not find any changes in glycaemic control [42, 43].

Particularly, T2D results in very high economic and social costs [1]. Due to the evidence of a mutual impact of T2D and SDB, the high proportion of SDB in patients with T2D, and the clinical relevance, monitoring for SDB might be profitable as this proportion of patients could benefit from SDB therapy.

Especially patients with typical symptoms like daytime sleepiness, heavy snoring or already occurring apnoeas should be tested for SDB [33, 44], because treatment of SDB with continuous positive airway pressure improves quality of life and daytime sleepiness, even in patients with mild SDB-related symptoms [9, 45]. Additionally, physicians treating patients with SDB should also consider the possibility of T2D and should initiate appropriate tests or measures [33].

The strengths of our study are the large sample size and high-resolution phenotyping of a T2D outpatient cohort with a comparable number of men and women (61%/*n* = 412 versus 39%/*n* = 267), thus enabling us to investigate effects specific to sex and using multivariable modelling. It expands on the knowledge from previous studies that were restricted to a clinically more severe diabetes status.

There are also some limitations that warrant discussion: first, although the use of portable respiratory devices instead of inpatient overnight polysomnography is well established and validated for assessment of SDB [18, 19], we cannot distinguish between obstructive and central sleep apnoea due to simplified monitoring. Second, left ventricular ejection fraction and heart failure were not evaluated in DIACORE, so we cannot report on their implications on the present study. Third, our sample might not be fully representative of an overall T2D patient group, because we did not standardize prevalence estimates to a specific population.

## 5. Conclusions

More than one-third of patients with T2D had moderate to severe sleep-disordered breathing, which was more common in men than in women. Higher age, higher BMI, and higher waist-hip ratio were associated with higher AHI. Modulators for higher AHI were similar in men and in women. Testing for SDB in patients with type 2 diabetes is recommended and vice versa.

## Figures and Tables

**Table 1:** Clinical characteristics of the 679 analysed subjects of the DIACORE baseline visit.

Characteristic	Women (*n* = 267)	Men (*n* = 412)	All (*n* = 679)
Age [years]	65 ± 10	66 ± 8	66 ± 9
BMI [kg/m^2^]	31.9 ± 6.0	30.3 ± 4.8	31.0 ± 5.4
Waist-hip ratio	0.9 ± 0.07	1.00 ± 0.06	0.96 ± 0.08
Waist circumference [cm]	98 ± 14	102 ± 18	101 ± 17
Triglycerides [mg/dl]	163 ± 80	177 ± 156	171 ± 131
HDL-C [mg/dl]	58 ± 16	50 ± 13	53 ± 15
T2D duration [years]	9.4 ± 7.5	10.6 ± 8.3	10.1 ± 8.0
HbA_1c_ [%]	6.8 ± 1.0	6.9 ± 1.2	6.8 ± 1.1
HbA_1c_ [mmol/mol]	50.8 ± 12.6	51.9 ± 10.4	50.8 ± 11.5
HOMA-IR^†^	6.3 ± 8.5	6.0 ± 6.7	6.1 ± 7.4
Systolic BP [mmHg]	136 ± 18	139 ± 18	138 ± 18
Metabolic syndrome [%]^∗^	83.5	68.8	74.6
Coronary artery disease [%]	9.7	28.4	21.1
Acute myocardial infarction [%]	6.0	15.8	11.9
Insulin [%]	24.0	28.6	26.8
Oral antidiabetics [%]	76.4	76.5	76.4
Sulfonylureas [%]	13.5	21.8	18.6
Incretins [%]	21.3	23.5	22.7

Data are expressed as percentage (%) for categorical variables and mean ± standard deviation for continuous variables. BMI = body mass index; HDL-C = high-density lipoprotein cholesterol; T2D = type 2 diabetes; BP = blood pressure; HbA_1c_ = glycated hemoglobin; HOMA-IR = homeostasis model assessment insulin resistance (fasting, use of long-acting insulin). ^†^ *n* = 413. ^∗^Metabolic syndrome is defined according to NCEP criteria [26].

**Table 2:** Clinical and SDB parameters of the 679 analysed participants at the DIACORE baseline visit by SDB class.

	No or mild SDB (AHI < 15)	Moderate SDB (AHI ≥ 15 to 29)	Severe SDB (AHI ≥ 30)	*p* value^∞^	Moderate versus no/mild SDB^§^	Severe versus no/mild SDB^§^
Number of patients, *n* (%)	451 (66%)	163 (24%)	65 (10%)			
Age [years]	64 ± 9	66 ± 8	67 ± 7	**0.008**	**0.030**	0.088
Female, *n* (%)	208 (46%)	43 (26%)	16 (25%)	**<0.001**	**<0.001**	**0.002**
BMI [kg/m^2^]	30.4 ± 5.1	31.3 ± 5.2	34.0 ± 6.8	**<0.001**	0.139	**<0.001**
Waist-hip ratio	0.95 ± 0.08	0.98 ± 0.07	1.00 ± 0.08	**<0.001**	**<0.001**	**<0.001**
Waist circumference [cm]	99 ± 16	102 ± 16	112 ± 15	**<0.001**	0.054	**<0.001**
Triglycerides [mg/dl]	166 ± 125	181 ± 155	187 ± 107	0.273	0.597	0.699
HDL-C [mg/dl]	54 ± 16	51 ± 14	50 ± 13	**0.016**	0.080	0.080
T2D duration [years]	10 ± 7	12 ± 10	9 ± 7	**0.001**	**0.001**	1.000
HbA_1c_ [%]	6.8 ± 1.1	6.9 ± 1.2	6.6 ± 0.7	0.111	0.867	0.340
HbA_1c_ [mmol/mol]	51 ± 7	52 ± 8	49 ± 5	0.111	0.867	0.340
HOMA-IR^†^	5.6 ± 5.9	6.7 ± 9.1	7.9 ± 10.9	0.137	0.607	0.242
Systolic BP [mmHg]	137 ± 18	140 ± 19	141 ± 17	**0.041**	0.135	0.179
Metabolic syndrome [%] ^∗^	71.8	78.4	84.6	**0.038**	0.290	0.079
Coronary artery disease [%]	16.0	23.3	20.0	0.101	0.083	0.235
Acute myocardial infarction [%]	10.9	13.5	15.4	0.448	1.000	0.882
Insulin [%]	22.6	37.4	29.2	**0.001**	**0.001**	0.771
Oral antidiabetics [%]	77.6	74.2	73.8	0.601	1.000	1.000
Sulfonylureas [%]	19.3	18.4	13.8	0.573	1.000	0.877
Incretins [%]	24.4	18.4	21.5	0.288	0.355	1.000
*SDB parameters*						
AHI [/h]	7 ± 4	21 ± 4	46 ± 12	**0.001**	**<0.001**	**<0.001**
ODI [/h]	7 ± 5	20 ± 5	41 ± 12	**0.001**	**<0.001**	**<0.001**
SpO_2_ < 90% [min]	73 ± 106	125 ± 122	120 ± 85	**0.001**	**<0.001**	**0.004**
SpO_2_ [%]	93 ± 2	92 ± 2	92 ± 2	**0.001**	**<0.001**	**<0.001**
Min SpO_2_ [%]	80 ± 12	75 ± 16	74 ± 15	**0.001**	**0.001**	**0.002**
Nocturnal breathing rate [/min]	15 ± 3	14 ± 3	12 ± 3	**0.001**	0.064	**<0.001**
Nocturnal heart rate [/min]	66 ± 9	66 ± 10	67 ± 10	0.667	1.000	1.000
ESS	5.1 ± 3.3	5.4 ± 3.6	5.9 ± 3.1	0.148	1.000	0.195
ESS ≥ 11, *n* (%)	29 (6.5)	10 (6.4)	6 (9.5)	0.661	1.000	1.000
Nocturia (≥3x/night) [%]	64 (14%)	25 (16%)	11 (17%)	0.785	1.000	1.000

Data are expressed as percentage (%) for categorical variables and mean ± standard deviation for continuous variables. ^∞^Difference of parameters between the three SDB groups assessed by chi-square tests for categorical variables and by ANOVA for continuous variables. ^**§**^Difference of parameters between two SDB groups assessed by post hoc analysis (Bonferroni). *p* < 0.05 was considered statistically significant and marked in bold. SDB = sleep-disordered breathing; BMI = body mass index; HDL-C = high-density lipoprotein cholesterol; T2D = type 2 diabetes; BP = blood pressure; HbA_1c_ = glycated hemoglobin; HOMA-IR = homeostasis model assessment insulin resistance (fasting, use of long-acting insulin); AHI = apnoea-hypopnoea index; ODI = oxygen desaturation index; SpO_2_ = peripheral oxygen saturation; ESS = Epworth Sleepiness Scale. ^†^ *n* = 413. ^∗^Metabolic syndrome is defined according to NCEP criteria [26].

**Table 3:** Linear regression analysis: factors associated with AHI in the total population, in 267 women and in 412 men.

Variable	*Univariable analysis*		*Multivariable analysis*	
	*B* (95% CI)	*p* value	*B* (95% CI)	*p* value
*Total population* (*n* = 679)				
Female sex	−4.75 (−6.74, −2.76)	**<0.001**	−3.54 (−6.06, −1.02)	**0.006**
Age [per 10 years]	1.70 (0.59, 2.81)	**0.003**	2.55 (1.40, 3.70)	**<0.001**
BMI [per 5 units]	2.99 (2.10, 3.88)	**<0.001**	3.48 (2.53, 4.44)	**<0.001**
Waist-hip ratio [per 0.1 unit]	4.48 (3.25, 5.71)	**<0.001**	2.35 (0.77, 3.94)	**0.004**
Systolic BP	0.08 (0.03, 0.13)	**0.004**	0.02 (−0.04, 0.07)	0.508
T2D duration	0.09 (−0.04, 0.21)	0.165	−0.004 (−0.13, 0.12)	0.952
*Women* (*n* = 267)				
Age [per 10 years]	1.92 (0.49, 3.35)	**0.009**	2.35 (0.83, 3.87)	**0.003**
BMI [per 5 units]	2.00 (0.87, 3.13)	**0.001**	2.05 (0.88, 3.21)	**0.001**
Waist-hip ratio [per 0.1 units]	3.24 (1.13, 5.35)	**0.003**	2.29 (0.18, 4.40)	**0.034**
Systolic BP	0.08 (0.01, 0.16)	**0.037**	0.03 (−0.05, 0.11)	0.446
T2D duration	0.06 (−0.13, 0.25)	0.527	−0.02 (−0.02, 0.17)	0.826
*Men* (*n* = 412)				
Age [per 10 years]	1.41 (−0.19, 3.01)	0.084	2.76 (1.09, 4.43)	**0.001**
BMI [per 5 units]	4.75 (3.45, 6.06)	**<0.001**	5.25 (3.73, 6.77)	**<0.001**
Waist-hip ratio [per 0.1 units]	4.92 (2.71, 7.12)	**<0.001**	1.59 (−0.77, 3.94)	0.185
Systolic BP	0.06 (−0.01, 0.14)	0.091	0.01 (−0.06, 0.09)	0.739
T2D duration	0.07 (−0.09, 0.23)	0.393	0.01 (−0.15, 0.17)	0.882

AHI = apnoea-hypopnoea index; *B* = unstandardized regression coefficient; 95% CI = 95% confidence interval; BMI = body mass index; T2D duration = duration of type 2 diabetes; BP = blood pressure.

**Table 4:** The prevalence and risk factors of SDB in T2D, comparison of existing studies.

Study, year (Ref.), country	Setting	Patients (*n*)	Source of patients	SDB diagnosis	Female, *n* (%)	Prevalence of SDB (%)	Modulators of SDB/higher AHI
Resnick et al., 2003 [10]^x^, USA	Multicenter	470	Sleep Heart Health Study (SDB as risk factor for the development of cardiovascular disease)	Polysomnography (Compumedics PS®, Melbourne, Australia)	254 (54)	24	AHI: age, BMI, male sex
Einhorn et al., 2007 [11], USA	Single center	279	Diabetes clinic (T2D)	SDB monitoring device (ApneaLink, ResMed Corp., San Diego, CA, USA)	133 (48)	36	SDB: age ≥ 62 yrs, male sex, BMI ≥ 30 kg/m^2^, snoring, reports of stopping breathing during sleep
Laaban et al., 2009 [13], France	Single center	303	Department of Diabetology (hospitalized for poorly controlled T2D)	SDB monitoring device (CID® 102, Cidelec, Angers, France)	147 (49)	29	—
Foster et al., 2009 [12], USA	Multicenter	305	Obese patients with T2D (Sleep AHEAD = Sleep Apnea in Look AHEAD—Action for Health in Diabetes)	Polysomnography (Compumedics®, Abbotsville, Australia)	183 (60)	53^#^	AHI/obstructive sleep apnoea ^∗^: waist circumference
Lam et al., 2010 [35], China	Single center	165	DM clinic	Polysomnography (Alice 5 Diagnostics System®, Respironics, Murrysville, PA, USA)	62 (38)	33^#^	AHI: age, BMI, diastolic BP, male sex^†^
Schober et al., 2011 [31], Germany	Multicenter	498	Primary care centers and medical department (endocrinology, metabolism)	SDB monitoring device (ApneaLink Oxi®, ResMed, Sydney, Australia)	237 (48)	41	—
Zhang et al., 2016 [34], China	Multicenter	880	Endocrinology ward (hospitalized)	SDB monitoring device (ApneaLink, ResMed, San Diego, CA, USA)	391 (44)	26	—
Kroner et al. (DIACORE), Germany	Multicenter	679	Outpatients	SDB monitoring device (ApneaLink, ResMed, Sydney, Australia)	267 (39)	34	AHI: age, BMI, waist-hip ratio

^∗^At cut-off AHI ≥ 5/h. ^†^ *n* = 162 (3 subjects with central sleep apnoea were excluded). ^#^Prevalence of obstructive sleep apnoea. ^x^It is not apparent which kind of diabetes sample was used. In other publications, it is interpreted as a sample of patients with type 2 diabetes [37]. Modulators of SDB/higher AHI were assessed in multivariable regression analysis. SDB = sleep-disordered breathing; BMI = body mass index; T2D = type 2 diabetes; AHI = apnoea-hypopnoea index; BP = blood pressure; RDI = respiratory disturbance index.

## Supplementary Materials

Supplementary Materials Supplement Figure S1: study flowchart showing the recruitment of the participants. SDB = sleep-disordered breathing. Supplement Table S2: different diagnostic devices and scoring criteria for respiratory events in similar studies.
