# Supplementary material for: Sex Differences in the Prevalence and Modulators of Sleep-Disordered Breathing in Outpatients with Type 2 Diabetes
Source: J Diabetes Res. 2018 Apr 1;2018:7617524. doi: 10.1155/2018/7617524 (PMC5901945; doi:10.1155/2018/7617524)
Supplement: Supplementary Materials — Supplement Figure S1: study flowchart showing the recruitment of the participants. SDB = sleep-disordered breathing. Supplement Table S2: different diagnostic devices and scoring criteria for respiratory events in similar studies. [file 7617524.f1.docx]

**Online Supplements**


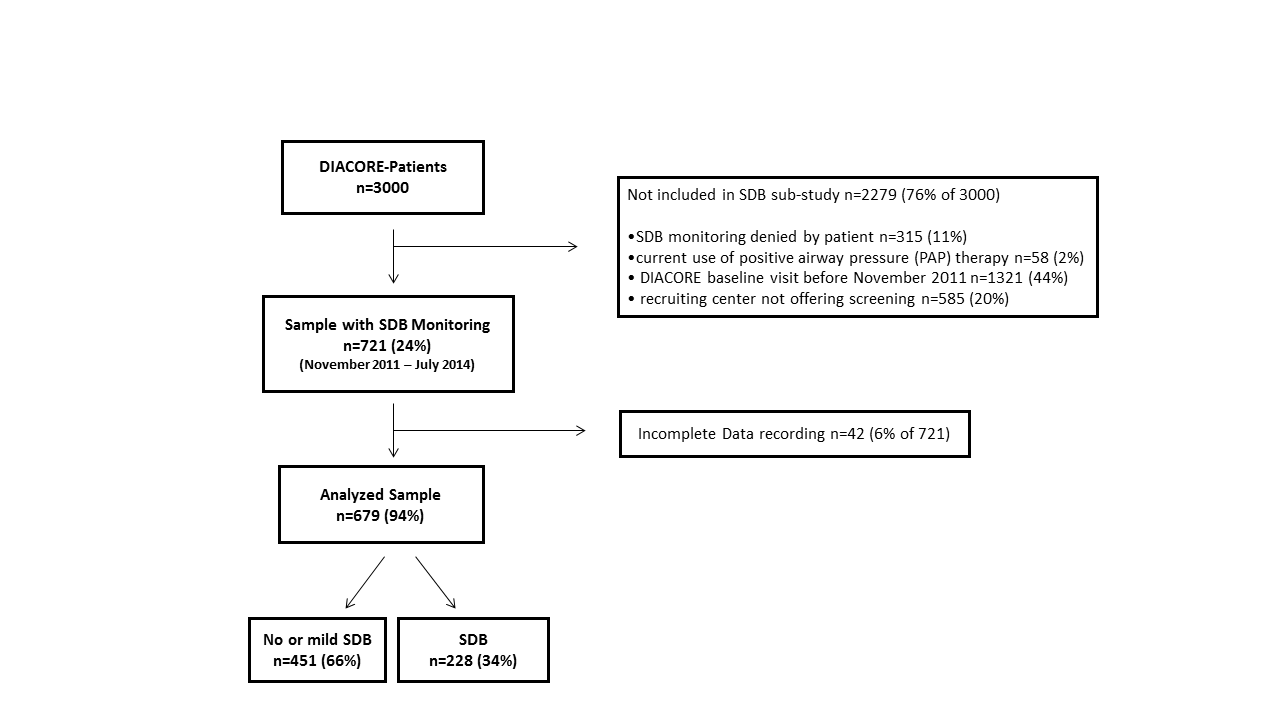


**Figure s1.** Study flowchart showing the recruitment of the participants.

SDB= sleep-disordered breathing

**Table s2**. Different diagnostic devices and scoring criteria for respiratory events in similar studies.

| **Study, Year (Ref.), Country** | **Diagnostic device** | **SDB Diagnosis** | **Scoring criteria for apneas** | **Scoring criteria for hypopneas** | **Scoring criteria for oxygen desaturations** |
| --- | --- | --- | --- | --- | --- |
| Resnick et al. (2003) [(17)](#_CTVL001082820a05f6a442f856312482b56a32e" \o "Resnick HE, Redline S, Shahar E et al. Diabetes and sleep disturbances: findings from the Sleep Heart Health Study. Diabetes Care. 2003 Mar; 26(3):702…), USA | Compumedics PS®  (Melbourne, Australia) | Polysomnography | Decrease of airflow/ respiratory effort to <25% of baseline amplitude for ≥10sec. | Decrease of airflow/ respiratory effort to 25-70% of baseline for ≥10sec. + desaturation | Decrease in oxygen saturation of ≥4%  (or ≥2%) |
| Einhorn et al. (2007) [(19)](#_CTVL001d5a2dcbf158447648e2cb6e7c06f069a), USA | ApneaLink®  (Resmed, San Diego, CA) | SDB-monitoring device | Decrease in airflow by ≥80% of baseline for ≥10sec. | Decrease in airflow by 50-80% of baseline for ≥10sec. + desaturation | Decrease in oxygen saturation of ≥4% |
| Laaban et al. (2009) [(18)](#_CTVL00195e52ef69000470a8011954ba4963e70), France | CID® 102  (Cidelec, Angers, France) | SDB-monitoring device | Complete cessation of airflow for ≥10sec. | Decrease of airflow by ≥50% for ≥10sec. + desaturation | Decrease in oxygen saturation of ≥4% |
| Foster et al. (2009) [(22)](#_CTVL00186d1bb7aa03542eba1f2a48d24ea2e83" \o "Foster GD, Sanders MH, Millman R et al. Obstructive sleep apnea among obese patients with type 2 diabetes. Diabetes Care. 2009 Jun; 32(6):1017-9.), USA | Compumedics®  (Abbotsville, Australia) | Polysomnography | Complete cessation of airflow for ≥10sec. | ≥ 30% reduction in airflow/respiratory effort for ≥10sec. +arousal/desaturation | Decrease in oxygen saturation of ≥4% |
| Lam et al. (2010) [(28)](#_CTVL001d6be6775d04a4ef19f59461ffd34844e), China | Alice 5 Diagnostics System® (Respironics, Murrysville, PA) | Polysomnography | Complete cessation of airflow for ≥10sec. | ≥30% reduction in airflow/respiratory effort for ≥10sec. + desaturation | Decrease in oxygen saturation of ≥4% |
| Schober et al. (2011) [(27)](#_CTVL001353af1c4738744019dfa81219f9f8ce8" \o "Schober AK, Neurath MF, Harsch IA. Prevalence of sleep apnoea in diabetic patients. Clin Respir J. 2011 Jul; 5(3):165–72.), Germany | ApneaLink Oxi®  (Resmed, Sydney, Australia) | SDB-monitoring device | Decrease in airflow by ≥80% of baseline for ≥10sec. | Decrease in airflow by 50-80% of baseline for ≥10sec. + desaturation | Decrease in oxygen saturation of ≥4% |
| Zhang et al. (2016) [(29)](#_CTVL001765d889b11104353b00663c2a826f967), China | ApneaLink®  (Resmed, San Diego, CA) | SDB-monitoring device | Decrease in airflow by ≥80% of baseline for ≥10sec. | Decrease in airflow by 50-80% of baseline for ≥10sec. + desaturation | Decrease in oxygen saturation of ≥4% |
| Kroner et al. (DIACORE), Germany | ApneaLink®  (Resmed, Sydney, Australia) | SDB-monitoring device | Decrease in airflow by ≥80% of baseline for ≥10sec. | Decrease in airflow by 50-80% of baseline for ≥10sec. + desaturation | Decrease in oxygen saturation of ≥4% |

SDB=sleep-disordered breathing
